# Mapping the interaction sites of human and avian influenza A viruses and complement factor H

**DOI:** 10.3389/fimmu.2024.1352022

**Published:** 2024-04-18

**Authors:** Iman Rabeeah, Elizabeth Billington, Béatrice Nal, Jean-Remy Sadeyen, Ansar A. Pathan, Munir Iqbal, Nigel J. Temperton, Peter F. Zipfel, Christine Skerka, Uday Kishore, Holly Shelton

**Affiliations:** ^1^ Pirbright Institute, Woking, United Kingdom; ^2^ Biosciences, College of Health, Medicine and Life Sciences, Brunel University London, Uxbridge, United Kingdom; ^3^ Aix-Marseille Université, CNRS, INSERM, CIML, Marseille, France; ^4^ Viral Pseudotype Unit, University of Kent, Chatham, United Kingdom; ^5^ Department of Infection Biology, Leibniz Institute for Natural Product Research and Infection Biology, Jena, Germany; ^6^ Institute of Microbiology, Friedrich Schiller University, Jena, Germany; ^7^ Department of Veterinary Medicine, United Arab Emirates University, Al Ain, United Arab Emirates; ^8^ Zayed Centre for Biomedical Sciences, U.A.E. University, Al Ain, United Arab Emirates

**Keywords:** complement, factor H, influenza A virus, avian influenza, human influenza, pseudotype, entry inhibitor

## Abstract

The complement system is an innate immune mechanism against microbial infections. It involves a cascade of effector molecules that is activated via classical, lectin and alternative pathways. Consequently, many pathogens bind to or incorporate in their structures host negative regulators of the complement pathways as an evasion mechanism. Factor H (FH) is a negative regulator of the complement alternative pathway that protects “self” cells of the host from non-specific complement attack. FH has been shown to bind viruses including human influenza A viruses (IAVs). In addition to its involvement in the regulation of complement activation, FH has also been shown to perform a range of functions on its own including its direct interaction with pathogens. Here, we show that human FH can bind directly to IAVs of both human and avian origin, and the interaction is mediated via the IAV surface glycoprotein haemagglutinin (HA). HA bound to common pathogen binding footprints on the FH structure, complement control protein modules, CCP 5-7 and CCP 15-20. The FH binding to H1 and H3 showed that the interaction overlapped with the receptor binding site of both HAs, but the footprint was more extensive for the H3 HA than the H1 HA. The HA - FH interaction impeded the initial entry of H1N1 and H3N2 IAV strains but its impact on viral multicycle replication in human lung cells was strain-specific. The H3N2 virus binding to cells was significantly inhibited by preincubation with FH, whereas there was no alteration in replicative rate and progeny virus release for human H1N1, or avian H9N2 and H5N3 IAV strains. We have mapped the interaction between FH and IAV, the *in vivo* significance of which for the virus or host is yet to be elucidated.

## Introduction

Influenza A viruses (IAVs) are enveloped viruses that carry a segmented, negative sense, single-stranded RNA genome. Human H1N1 and H3N2 IAVs cause regular seasonal outbreaks in the human population where the infection can result in severe clinical outcomes in children, elderly, pregnant women, and immune compromised individuals. The public health burden of these annual outbreaks is significant with up to 650,000 excess deaths estimated annually by the World Health Organisation (WHO) from influenza infections (1). In addition, a vast array of different IAV subtypes circulate in the natural reservoir population of wild aquatic birds, and sporadically, these avian IAVs can cross the host species barrier and result in infection of humans, often with high case fatality rates, such as those caused by H7N9 or H5N1 (2–4).

The complement system is one of the very first innate immune responses mounted by vertebrates against pathogens. The complement system is activated using three different pathways, namely the classical, the lectin and the alternative pathways. Although different in terms of their first recognition subcomponent, all the three complement pathways converge on the generation of C3 and C5 convertase, production of anaphylatoxins C3a and C5a, and lytic membrane attack complex (MAC). Consequently, the complement activation helps clear the viruses via virolysis, pro-inflammatory cytokine production and potentiation of the adaptive immunity (5, 6). It has been demonstrated that the complement system can control IAV infections via the classical pathway, whereby virus-specific, or non-specific natural IgG antibodies bind to the virus allowing its association with C1 of the classical pathway (7, 8). The subsequent deposition of complement components onto the virus surface leads to virus aggregation and inhibition of receptor binding, and thus, viral neutralisation (9, 10). The lectin pathway acting through the binding of either mannan-binding lectin (MBL) or L-ficolin to IAV surface Haemagglutinin (HA) protein, together with the alternative pathway, has also both been shown to contribute to the protection of the host from IAV (11, 12).

In order to successfully replicate and transmit, many viruses have evolved complement evasion strategies (13). Incorporation of complement regulators into virion structures, such as the central complement inhibitors, CD55 and CD59, which are found in the HIV membrane envelope is one such strategy (14). Virus mimicry of complement regulators also occurs, for example, the gC of Herpes Simplex Virus 1 mimics the action of CD55 or CD59, inhibiting the C3b molecule central to the complement activation (15, 16). Additionally, viruses have been shown to perturb complement protein expression levels. In Hepatitis C virus infected cells, there is a down-regulation of C2, C4, C3 and C9 expression which results in reduced complement activation (17–19). IAV is known to incorporate CD59 into its envelope in order to protect against MAC-mediated lysis by preventing C9 polymerisation (20). In addition, the Matrix (M1) protein of IAV binds to the recognition subcomponent C1q, which prevents classical pathway activation. However, at what point in the virus lifecycle, the M1 protein, which is internal to the virus structure, gets to contact C1q is not known (21).

Factor H (FH) is a large, 150 kDa plasma glycoprotein, which acts as a negative regulator of the alternative pathway (22). Unlike the classical and lectin pathways, it does not require a specific binding event to get activated; instead, it is initiated by spontaneous hydrolysis of C3. FH regulates this process by binding to C3b, which inhibits the formation of the critical C3 convertase enzyme. FH is also a co-factor for factor I, a protease that cleaves C3b into an inactive form (iC3b). These actions by FH act to protect the hosts’ self-structures from this non-directed alternative pathway (23). Several pathogens bind to host FH as an evasion mechanism to protect against alternative pathway mediated lysis (24, 25). Pathogenic bacteria including *Borrelia burgdorferi*, *Staphylococcus aureus*, *Streptococcus pneumoniae*, *Neisseria meningitidis* and *Neisseria gonorrhoeae* code for proteins that bind to FH, thus providing mimicry of the hosts self surfaces, which leads to an increased survival of the pathogens (24).

Viruses can also directly bind FH to evade the complement system. The NS1 protein of the Flavivirus, West Nile Virus (WNV), has been shown to interact with human FH (26). The WNV NS1 protein is a secreted glycoprotein which can then bind back to the surface of cells, so its interaction with FH may reduce the recognition of infected cells by the complement leading to pathogen survival. Zaiss et al. have demonstrated that the capsid proteins of Adeno-associated virus (AAV) can also interact with human FH, which allows the inhibition of C3 convertase on the virion surface (27). In addition, human IAV strains have been shown to interact directly with FH (28) and modulate differentially infection of lung epithelial cell line, A549 cells, by IAV strains: this effect of factor H binding to IAV subtypes was assayed in a way that did not activate complement (i.e. in serum-free conditions), suggesting that FH can have a complement-activation independent functions against IAV.

In this study, we confirm that there is a direct interaction of purified human FH with human and avian subtypes of IAVs. We demonstrate that human FH binds to the IAV envelope HA glycoprotein using its common pathogen binding domains, which are also involved in FH binding to bacterial proteins. The interaction site of FH on the HA of human H1 and H3 subtypes is in the vicinity of the receptor binding site and the consequence of this is an initial inhibition of viral entry to target cells when FH is present. However, there is a more extensive FH binding footprint on the H3 HA compared to the H1 HA, and this results in the modulation of multicycle replication that is IAV strain-dependent.

## Materials and methods

### Cells

A549 and MDCK cells (Cell Culture Central Services Unit, Pirbright Institute, UK) were grown in Complete Medium, DMEM (Gibco-Invitrogen, Inc.), supplemented with 10% (v/v) foetal bovine serum (FBS) (Biosera, Inc.), 1% (v/v) penicillin/streptomycin (Sigma-Aldrich) and 1% (v/v) non-essential amino acids (Sigma-Aldrich), and maintained at 37°C in 5% CO_2_.

### Viruses

Various Influenza A viruses used in this study are listed in **Table 1**.

**Table 1:** Viruses used in this study.

IAV strain	Subtype	Source
A/Hong Kong/1774/99	H3N2	Leo Poon, University of Hong Kong
A/Hong Kong/4801/14	H3N2	NIBSC (NYMC-X-263)
A/England/195/09	H1N1	Wendy Barclay, Imperial College London (29)
A/Michigan/45/2015	H1N1	NIBSC (NYMC X-275)
A/chicken/Pakistan/UDL01/08	H9N2	Reverse genetics (30)
A/duck/Singapore/3/97	H5N3	Munir Iqbal, Pirbright Institute

Human IAV strains were propagated in MDCK cells in the presence of 1 µg/ml TPCK trypsin (Sigma) and harvested after 72 hours. Avian influenza strains were propagated in specific pathogen free (SPF) 10-day old embryonated chicken eggs (VALO BioMedia, Germany), and the allantoic fluid was harvested after 48 hours. Purification of IAVs was performed via ultra-centrifugation using a 30% sucrose cushion. Briefly, virus solution was centrifuged at 10,000 rpm at 4°C for 30 minutes in a chilled SW32Ti rotor (Beckman Coulter) to remove debris; this was followed by another round of ultracentrifugation using a 30% cold sucrose solution at 25,000 rpm for 90 minutes. The viral pellet was recovered and resuspended in PBS. Viral titre was determined via a plaque assay involving MDCK cells, as previously described (30), or by TCID50 assay. Briefly, TCID50 was performed by diluting virus in half Log_10_ series across a 96-well tissue culture plate in serum-free DMEM. 5 x 10^5^ MDCK cells per ml were prepared in serum free DMEM containing 1% v/v penicillin/streptomycin (Sigma-Aldrich) and 1 µg/ml TPCK Trypsin (Sigma). 100 µl of MDCK cells were added to each well and incubated at 37°C under 5% CO_2_ for 3 days. Cells were fixed with ice cold 50:50 methanol:acetone for 20 minutes and stained with 0.1% crystal violet solution containing methanol. Cytopathic effect (CPE) was recorded for each well and TCID50 calculated by the Reed-Muench method (31).

### Lentiviral pseudotyped particles

MLV particles carrying a firefly luciferase expression plasmid were pseudotyped with IAV HA alone, or IAV HA and NA, the VSV-G protein, or without an envelope protein. Pseudotyped lentivirus particles were produced in HEK293T cells as described earlier (32, 33), and titrated by quantification of relative light units produced by luciferase activity in the transduced HEK293T cells (**Table 2**).

**Table 2:** Envelope proteins used to produce pseudotyped lentiviral particles.

Viral strain	IAV Subtype	Envelope receptors
A/Udorn/307/1972	H3N2	HA
A/California/7/2004	H3N2	HA
A/Texas/1/1977	H3N2	HA
A/Solomon Island/3/2006	H1N1	HA
A/England/195/2009	H1N1	HA
A/Hong Kong/33982/2009	H9N2	HA
A/FPV/Rostock/1934	H7N1	HA
A/Shanghai/02/2013	H7N9	HA
A/Vietnam/1194/2004	H5N8	HA
A/Vietnam/1194/2004	H5N8	HA+NA
De-envelope protein		–
Vesicular stomatitis virus		G

### Purification of human FH protein

Human FH was purified from pooled human plasma (TCS Biosciences Ltd) via a three-step affinity chromatography procedure (34). The first step involved removal of plasmin/plasminogen from plasma. A L-Lysine – Sepharose column [1 g of Cyanogen bromide (CNBr)-activated Sepharose 4B (Sigma)] was added to 15 ml of 100 mM NaHCO_3_ buffer, pH 8.9 with 2 g of L-lysine monohydrochloride (Fisher Scientific). The column was equilibrated with binding buffer [100 mM sodium phosphate, 150 mM NaCl, 15 mM EDTA, pH 7.4)], and then 50 ml of human plasma was applied at the flow rate of 1 ml/minute. The column was washed with 100 ml of binding buffer, the flow-through was collected, and then dialysed against buffer containing 25 mM Tris–HCL, 140 mM NaCl, 0.5 mM EDTA, pH 7.4.

The next step involved removal of IgG binding proteins from plasma. Purified human IgG (20 mg) in PBS was immobilized on to CNBr-activated Sepharose (10 mg IgG/ml packed Sepharose). The reactive sites in the Sepharose were blocked by the addition of 20 volumes of 100 mM Tris-HCl, 150 mM NaCl, pH 8.5 for 2 hours. The column was equilibrated in running buffer (25 mM Tris–HCl, 140 mM NaCl, 0.5 mM EDTA, pH 7.4). The plasmin/plasminogen free human plasma was then passed through the IgG–Sepharose column in running buffer, and the column was washed with 100 ml of running buffer. The flow-through was again collected.

The last chromatographic step involved use of anti-human FH (OX23) monoclonal antibody (35). Purified anti-FH OX23 antibody (8 mg; MRC Immunochemistry Unit, Oxford) from mouse hybridoma cell line culture supernatant was immobilized on CNBr-activated Sepharose (2 mg/ml packed Sepharose). The column was equilibrated in running buffer, and then the plasma depleted of plasmin/plasminogen and IgG binding proteins was passed through the antibody affinity column. The column was washed extensively with 100 ml of running buffer and then FH protein was eluted in 1 ml fractions using 3 M MgCl_2_. Fractions containing FH were pooled and dialysed against PBS, and stored at -20°C in aliquots. The purity of FH was assessed by 10% w/v SDS-PAGE (**Supplementary Figure 1A**).

### HA peptides

H1 and H3 peptides were custom made by Alta Bioscience Ltd, based on the sequence of H1N1 A/England/195/09 and H3N2 A/Hong Kong/1774/99. Both HA sequences were covered by 56 peptides; 55 of the peptides for each HA were 20 amino acid long with an overlap along the protein sequence of 10 amino acids; the final peptide sequences were 16 (H1) or 15 (H3) amino acids in length. Each peptide had an N-terminal biotin tag and an amide at the C-terminus. The sequences of the synthetic peptides are listed in **Supplementary Table 1**.

### ELISA

The interaction of FH or FH CCP fragments with IAV and pseudotyped lentivirus with was assessed by ELISA. Purified IAV (1 x 10^4^) or 10^4^ relative light units (RLU) of pseudotype lentivirus were adsorbed onto 96-well Maxisorp microtitre plates (Fisher Scientific) in carbonate-bicarbonate (CBC) buffer, pH 9.6 (Sigma) overnight at 4°C. Plates were then washed with PBST (PBS with 0.1% Tween-20). Wells were blocked for 1 hour at room temperature with 5% w/v BSA in PBS (blocking buffer). Purified FH or FH CCP fragments diluted in PBS were added at various known concentrations and incubated for 2 hours at room temperature. Wells were washed with PBST, and primary antibody, either the OX24 monoclonal anti-FH (purified from mouse hybridoma supernatant; MRC Immunochemistry Unit, Oxford), or a polyclonal goat anti-human FH (Complement Technology), diluted in blocking buffer, were added to the wells and incubated for 1 hour at room temperature. After washing in PBST, secondary antibody, rabbit anti-mouse-HRP (DAKO) or rabbit anti-goat (Abcam), diluted in blocking buffer, was applied for 1 hour at room temperature. Following incubation, wells were washed in PBST and the color was developed using 3,3′,5,5′-Tetramethylbenzidine (TMB) substrate (Sigma). The reaction was stopped using 2 N H_2_SO_4_ (Sigma). The absorbance was read at 450 nm using microplate Reader ELX 808 (Bio-Tek).

### Virus cell entry assay

H1N1 A/England/195/09 or H3N2 A/Hong Kong/1774/99/IAV at a MOI of 1 was preincubated with or without 100 µg/ml of purified FH for 1 hour at 37°C. Virus was then added to A549 cells and allowed to bind cells for 1 hour at 37°C. Cells were washed to remove unbound viruses, and then incubated at 37°C with 5% CO_2_ in serum free DMEM containing 1% penicillin/streptomycin (Sigma) and 1 µg/ml TPCK Trypsin (Sigma) for either 4 hours or 8 hours. At the appropriate timepoint, cells were either fixed with ice cold 50:50 methanol: acetone for 5 minutes at room temperature, or lysed by scraping into buffer containing 1% Triton X 100, 20 mM Tris-HCl, 150 mM NaCl, 1 mM EDTA, together with 1% anti-proteases cocktail (Thermo Scientific). Fixed cells were immune stained for viral NP protein using mouse anti-NP (Pirbright Institute) and goat anti-mouse HRP conjugate, followed by development with AEC Substrate Chromogen (DAKO); images were acquired via EVOS XL Core Cell Imaging system (Invitrogen). Lysed cells were run on a 10% v/v SDS-PAGE under reducing conditions and western blotting was carried out. The blot was probed using mouse monoclonal anti-M1 (Bio-Rad) and goat anti-mouse IRDye 800CW (LiCor), and visualized using the Odessey Imaging system (LiCor).

### Virus infection assay

A549 cells were infected with a MOI 0.1 of H1N1 (A/England/195/09) or H3N2 (A/Hong Kong/1174/99) IAV that were preincubated with various concentrations of FH (0, 5, 50 or 100 µg). Alternatively, A549 cells were infected with a MOI 0.1 of H9N2 (A/chicken/Pakistan/UDL01/08) or H5N3 (A/duck/Singapore/3/97) IAV that were preincubated with 100 µg of FH. Cells were then incubated at 37°C under 5% CO_2_ in serum free DMEM containing 1% penicillin/streptomycin (Sigma) and 1 µg/ml TPCK Trypsin (Sigma) for 24 hours, after which the cell supernatants were removed from infected A549 cells and titrated for infectious virus via TCID50 assay.

## Results

### Human FH binds to human and avian influenza A viruses

**Figure 1:**
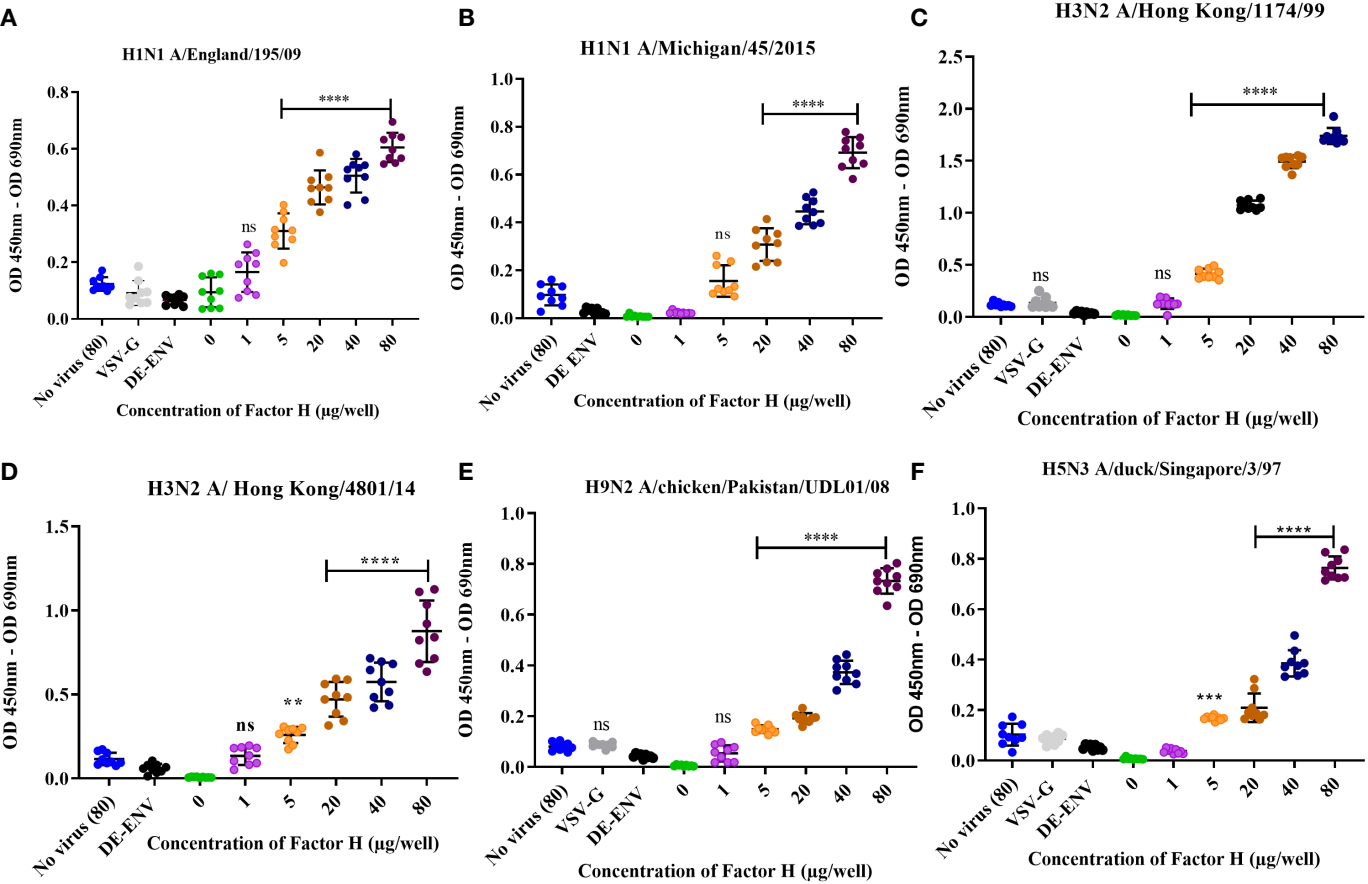
Human factor H protein binds to Influenza A viruses. An ELISA was carried out to assess the direct interaction between FH and IAV strains **(A)** H1N1 A/England/195/09, **(B)** A/Michigan/45/2015, **(C)** H3N2 A/Hong Kong/1174/99, **(D)** H3N2 A/Hong Kong/4801/14, **(E)** H9N2 A/chicken/Pakistan/UDL01/08 and **(F)** H5N3 A/duck/Singapore/3/97. Concentrations of 0, 1, 5, 20, 40 and 80 µg/well of purified FH were tested with 10^4^ pfu/well of IAV alongside the controls of 80 µg FH with no virus (No virus (80), lentivirus particles with VSV-G in the envelope (VSV-G) and lentivirus particles generated with no envelope protein (DE-ENV). FH binding was detected by anti-FH monoclonal antibody (OX24). Individual values from three independent experiments having three replicates per experiment are shown with mean OD 450nm – OD 690nm and error bars indicating standard deviation. Significantly increased binding was calculated by One way ANOVA and *post hoc* Dunnetts multiple comparison test to the control group of no virus (80), **p ≤ 0.01, ***p ≤0.001 and ****p ≤0.0001. ns, not significant.

To test the interaction between human FH and IAV, purified FH and IAV strains were produced (**Supplementary Figure 1**). Using an ELISA assay, we tested the binding of a range of concentrations (0 – 80 µg/well) of affinity purified human FH protein to 10^4^ pfu of human and avian IAV viruses (**Figure 1**). Two strains of human H1N1 virus (A/Michigan/45/2015 and A/England/195/2009) and two human H3N2 viruses (A/Hong Kong/1774/1999 and A/Hong Kong/4801/14) were tested. Significant binding (p < 0.05) was seen for all four human viruses at the FH concentration of 40 µg/well (**Figures 1A–D**). Control groups included no FH (0 µg/well), no virus, lentivirus particles with no envelope proteins (ENV), and lentivirus particles with VSV-G protein embedded in the membrane, the latter three control groups had 80 µg/well of FH applied to them. Since avian viruses can also sporadically cross the species barrier to infect humans, we also examined possible interaction between human FH and avian influenza viruses. Similar to the human IAV strains, FH protein (40µg/well) bound significantly to H9N2 (A/chicken/Pakistan/UDL01/2008) as well as H5N3 (A/duck/Singapore/3/97) IAV (**Figures 1E, F**).

### Human FH binds IAV via its common pathogen binding surfaces

**Figure 2:**
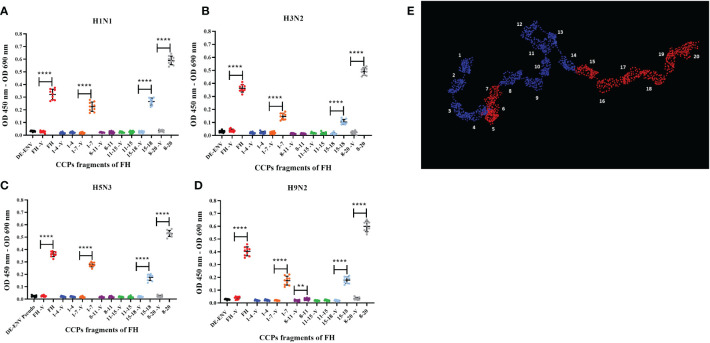
Human FH binds IAV strains via its CCP domains 5-7 and 15-20. 10^3^ pfu/well of IAV; **(A)** H1N1 A/England/195/09, **(B)** H3N2 A/Hong Kong/1174/99, **(C)** H5N3 A/duck/Singapore/3/97 and **(D)** H9N2 A/chicken/Pakistan/UDL01/08 were used in an ELISA using 5 µg/well of purified FH protein (FH) or CCP fragments of FH, (CCP 1-4, 1-7, 8-11, 11-15, 15-18 and 8-20). Each fragment was assessed against its own controls of 5 µg FH with no virus (-V) and lentivirus particles generated with no envelope protein (DE-ENV). FH binding was detected by polyclonal goat anti-human FH. Individual values from three independent experiments carrying three replicates per experiment are shown with mean OD 450nm – OD 690nm and error bars indicating standard deviation. Significant binding was calculated by unpaired two-way T-test to the CCP fragment control group of -V, **p ≤ 0.01 and ****p ≤0.0001. **(E)** PyMOL generated structure from 1HAQ protein database entry of human FH, 20 CCP domains numbered and domains in red indicated predicted IAV interaction sites, CCP5-7 and CCP15-20 from the ELISA data.

A range of pathogens including bacteria, fungi and viruses have been shown to interact with human FH, predominantly via its CCP5-7 and CCP19-20 modules. These CCP modules also encompass the known binding sites for host cell ligands (36). Binding of pathogens to these sites does not interfere with FH interaction with C3b; thus, it does not functionally compromise the ability of FH to downregulate the complement alternative pathway (36). Using recombinant fragments corresponding to different portions of human FH protein (37, 38), we mapped the interaction of human and avian IAV strains using the ELISA (**Figures 2A–D**). All CCP fragments were detected by the polyclonal goat anti-human FH antibody used in ELISA (**Supplementary Figure 2A**). We found that all four IAV strains bound to the FH CCP8-20 fragment with the greatest signal; significant binding was observed for the FH fragments encompassing CCP1-7 and CCP15-18. In addition, for the H9N2 virus, we found significant interaction with the CCP8-11 fragment but not for the H1N1, H3N2 or H5N3 strains. There was no significant binding to any of the IAV strains by FH fragments containing CCP1-4 or CCP11-15 which suggested that the footprint for IAV on the FH protein could be refined to CCP5-7 and CCP15-20 (**Figure 2E**).

### Human FH binding to IAV is mediated via the HA viral envelope glycoprotein

**Figure 3:**
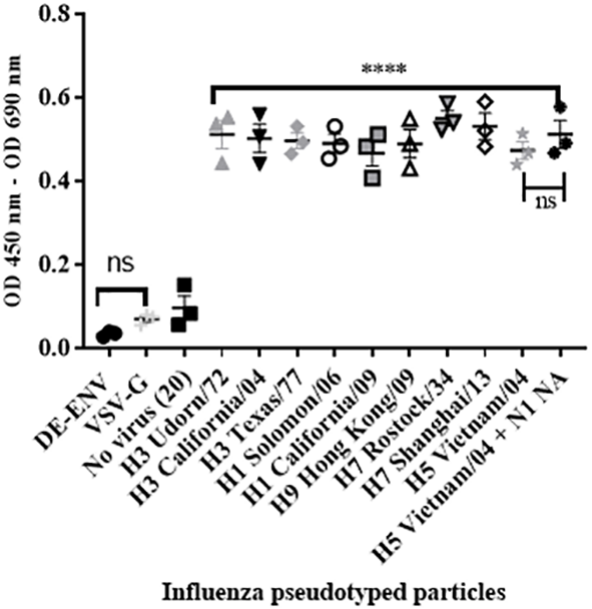
HA protein mediates interaction between human FH and IAV. 10^4^ RLU/well of lentiviruses pseudotyped with various envelope proteins were used in an ELISA with 20 µg/well of purified FH alongside the controls of 20 µg/well FH with no virus [No virus (20)], lentivirus particles with VSV-G in the envelope (VSV-G) and lentivirus particles generated with no envelope protein (DE-ENV). FH binding was detected by anti-FH monoclonal antibody (OX24). Mean OD 450nm – OD 690nm values with three independent experiments carrying three replicates per experiment are displayed with error bars, indicating standard error of the mean. Significant binding was calculated by One-way ANOVA and *post hoc* Dunnetts multiple comparison test to the control group of No virus (20); **** p ≤0.0001. ns, not significant.

To confirm that the interaction between human FH and IAV was mediated via the viral surface glycoprotein Haemagglutinin (HA), we performed the ELISA using lentiviruses pseudotyped with HA protein of different IAV subtypes. These lentiviruses only express the IAV HA protein on the surface of the virion. We tested a panel of human H1 and H3 HA proteins and avian H9, H7 or H5 HA, all of which bound the FH significantly compared to the controls (**Figure 3**), which was the lentivirus pseudotyped with the vesicular stomata virus (VSV) surface protein G. In addition, we tested a lentivirus pseudotyped with both H5 HA and N1 NA proteins from avian IAV and found that the addition of NA into the viral envelope did not significantly alter the binding of the FH to virus particle compared to the particle displaying H5 HA alone (**Figure 3**).

### FH binding footprint on HA is in the region of the receptor binding site

**Figure 4:**
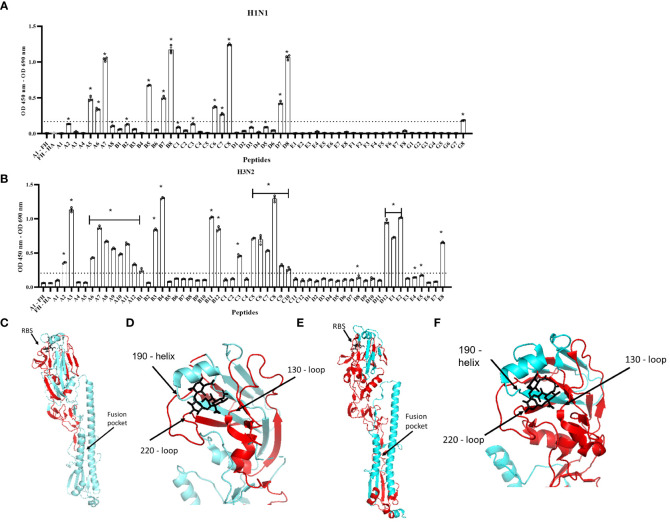
Human FH binds to the receptor binding region and fusion peptide pocket of IAV HA proteins. 5 µg/well of purified FH was used in ELISA assay with 2.5 µg/well of biotinylated 20-mer overlapping HA peptides. **(A)** H1N1 (A/England/195/09) and **(B)** H3N2 (A/Hong Kong/1774/99). Binding of the peptides to FH shown as mean OD 450nm – OD 690nm values are displayed with error bars indicating standard error of the mean. Negative controls whereby A1 peptide with no FH (A1 – FH) and FH with no HA peptide applied (FH – HA) were carried out alongside. Significant binding was calculated by One-way ANOVA and *post hoc* Dunnett’s multiple comparison test to the control group of FH – HA; *p ≤0.0001. Dashed line indicated mean difference from FH – HA of greater than 0.15 OD. The panel is representative of two independent experiments carrying three replicates per experiment. **(C, D)** H1 HA structure (A/California/7/09) (protein data bank 3UBN) **(E, F)** H3 HA structure (A/Finland/486/2004) (protein data bank 2YP3) indicating FH binding footprint (red), sialic acid (black) in the receptor binding site (RBS). Major structural features RBS and fusion peptide pocket are indicated, and structural RBS loops 130, 180 and 220 marked. Images were generated using the PyMOL Molecular Graphics System, Version 2.0 Schrödinger, LLC.

To map the footprint of FH binding to the H1N1 (A/England/195/09) and H3N2 (A/Hong Kong/1774/99) HA proteins, we used a panel of N-terminal biotinylated 20mer peptides from the HA proteins that overlapped by 10 amino acids with the preceding and following peptides (**Supplementary Tables 1**, **2**). The peptides solubility was confirmed by dot blot using HRP conjugated Streptavidin (**Supplementary Figures 2B, C**). An ELISA was performed whereby the biotinylated peptides were immobilized on streptavidin-coated microtiter wells and the binding of FH to the peptides was examined using the anti-FH monoclonal antibody OX24 (**Table 1**; **Figure 4**). We identified the binding footprint of FH on both the H1 and H3 proteins. Similarly, for both HAs, FH bound to peptides that made up the receptor binding site, with interactions with both the amino acids in HA1 of the 130-loop and 220-loop. This suggests that binding of FH to HA might interfere with its ability to interact with the virus receptor, i.e. sialic acids, at the surface of target cells (**Figures 4C–F**). There were more peptides spanning the H3 HA protein that bound to FH compared to the H1 HA (**Figure 4**). Interestingly, peptides which form the bottom of the fusion peptide pocket in HA2 of the H3 HA structure, but not the H1 HA, also interacted with FH. It is interesting to note that two of the peptides that interact the most with FH are D8 on H1 and C8 on H3 and those are slightly upstream the trypsin cleavage sites (QSR/QTR). These peptides are conserved between H1 and H3. It is possible that the cleavage by trypsin can be inhibited by FH binding. If this is the case, then endocytosis may occur but fusion may be impaired, unless FH is dissociated at low pH and cathepsin proteases cleaves HAs in endosomes.

### Human FH inhibits cell entry by human IAVs

**Figure 5:**
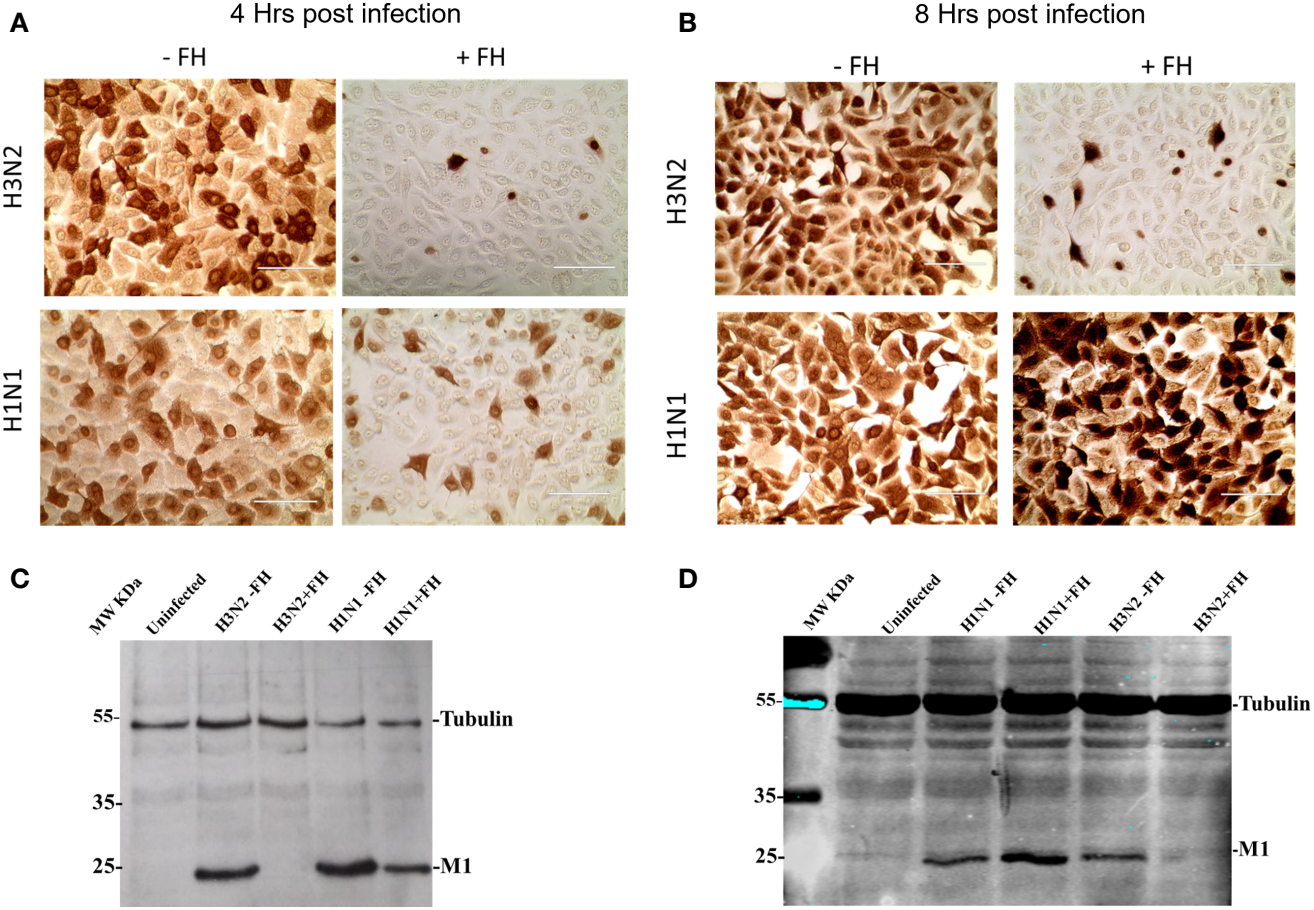
Pre-incubation of FH with human IAV strains alters virus entry to human lung cells. A549 cells were infected with a MOI 1 of H1N1 (A/England/195/09) or H3N2 (A/Hong Kong/1174/99) IAV preincubated with (+FH) or without FH (-FH). At 4 hours **(A)** or 8 hours post infection **(B)**, cells were fixed and immune-stained with anti-NP antibody (brown staining). At 4 hours **(C)** or 8 hours post infection **(D)**, cells were lysed and cell lysate was subjected to reducing SDS-PAGE. Proteins were western blotted, and the membrane probed using anti-M1 to detect IAV Matrix protein and anti-β tubulin as a loading control. Pre-stained MW protein marker was used as a reference (Thermo Scientific).

The HA protein of IAVs contains the sialic acid receptor binding site that mediates viral attachment to host cells. In addition, HA possesses a pH activated fusion peptide which facilitates cell membrane fusion between the IAV virion and cellular endosomal membranes to permit genome uncoating. Cleavage of HA by host proteases into HA1 and HA2 and conformational change of HA at low pH enable the extension of HA2 and insertion of its N-terminal fusion peptide into host membranes to facilitate envelope fusion. We hypothesized that binding of HA protein to human FH may affect the entry process of IAV during infection of target cells. This idea was tested by pre-incubating human IAVs with FH prior to a single round of infection in human lung cell line, A549, at a MOI of 1. At 4 and 8 hours post infection, the infected A549 cells were either fixed with ice cold 50:50 Methanol: acetone, or lysed. Fixed cells were immune-stained with an anti-nucleoprotein (NP) antibody (**Figures 5A, B**) whereas cell lysates were separated by denaturing and reducing SDS-PAGE, transferred for western blotting, and then probed with an anti-Matrix (M1) antibody (**Figures 5C, D**). At 4 hours post infection, we found that both human H1N1 (A/England/195/09) and H3N2 (A/Hong Kong/1774) IAV that were preincubated with FH prior to infection showed reduced entry to A549 cells by both immune-staining and western blot analysis compared to IAV that were not pre-incubated with FH (**Figures 5A, C**). The entry inhibition was greater for the human H3N2 strain compared to the H1N1 with almost a complete block observed in the case of H3N2. When we analyzed the infection of the A459 cells at 8 hours post infection for H3N2, we again observed this near complete inhibition of entry (**Figures 5B, D**). Interestingly, for the H1N1 IAV strain, we observed an enhancement of the amount of viral protein in the cells at 8 hours post infection when the H1N1 virus was pre-incubated with FH protein (**Figures 5B, D**). For the cell loading control, we used β-tubulin, which did not appear to change between the FH treated and untreated infected cells. Thus, the enhancement in IAV protein at 8 hours for H1N1 is not due to more cells being present in the samples.

### Interaction of FH with H3N2 IAV inhibits viral replication in A549 cells

**Figure 6:**
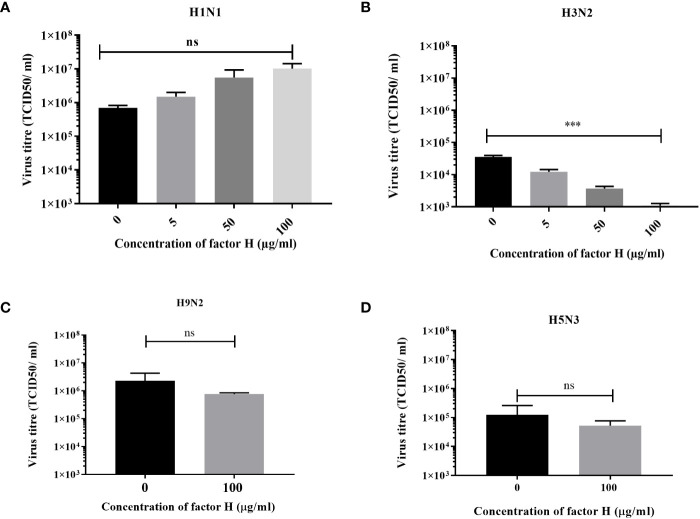
Preincubation of FH with H3N2 IAV inhibits replication in A549 cells. A549 cells were infected with a MOI 0.1 of **(A)** H1N1 (A/England/195/09) or **(B)** H3N2 (A/Hong Kong/1174/99) IAV that had been preincubated with various quantities of FH (0, 5, 50 or 100 µg). A549 cells were infected with a MOI 0.1 of **(C)** H9N2 (A/chicken/Pakistan/UDL01/08) or **(D)** H5N3 (A/duck/Singapore/3/97) IAV that were preincubated with 100 µg of FH. 24 hours post infection, the cell supernatants were harvested from infected A549 cells and titrated for infectious virus by TCID50 assay. The mean viral titre (TCID50/ml) is plotted in each panel with error bars indicating standard deviation. Unpaired, two-tailed t-test to 0 µg/ml FH was performed; ***p ≤0.001. ns, not significant.

We used a low MOI multi-cycle growth assay to determine whether the interaction between FH and IAV strains, which affected the entry of human IAV in A549 cells (**Figure 5**), altered the ability of the viruses to replicate and release progeny from these cells. The virus titres measured at 24 hours post infection demonstrated a strain specific alteration in virus replication (**Figure 6**). For the human strain H1N1 (A/England/195/09), there was a moderate effect on the titres collected from the A549 cells following preincubation of the virus with various concentrations of FH. In contrast, the released human H3N2 (A/Hong Kong/1774/99) virus titre was significantly lower at all tested concentrations of FH. We conclude that that less progeny viruses are produced following the first round of replication from FH-bound H3N2 viruses (**Figure 5**), and therefore, less cells are infected by these H3N2 progeny viruses. Both H9N2 (A/chicken/Pakistan/UDL01/08) and H5N3 (A/duck/Singapore/3/97) IAV titres were not affected by preincubation with 100 µg/ml of FH.

## Discussion

Factor H (FH) is a negative regulator of the complement alternative pathway. It functions to protect “self” cells from lysis by the complement system. Multiple pathogens including bacteria and viruses have the capacity to recruit FH to their surfaces as a complement evasion strategy (22). In this study, we validated the earlier report that FH can bind to human IAVs of the H1N1 and H3N2 subtype (28). In addition, we expand the observation to demonstrate that human FH is also able to bind avian influenza viruses of the H9 and H5 subtypes suggesting that the interaction is conserved amongst IAV subtypes. FH is abundant in plasma at concentrations ranging from 116 µg - 562 µg/ml (22). However, there is evidence that FH is present on mucosal surfaces and in the airway lumen as a result of local production by epithelial cells and leakage from the plasma under inflammatory conditions; this concentration is around 10% of plasma levels (10µg - 50µg/ml) (39, 40). Thus, FH is also available for interaction during natural IAV infection at the mucosal airway surfaces. The interactions we observed were significant at the physiological concentration of FH, down to 50µg/ml (5µg/well) (**Figure 1**) for most strains tested, H1N1 A/Michigan/45/2015 being the only exception that showed significant binding at 200µg/ml (20µg/well) (**Figure 1B**).

FH is composed of 20 complement control protein (CCP) modules (1-20), with CCP 1-4 known to be involved in C3b binding, decay acceleration of C3 convertase and as a cofactor for factor I. Two other C3b binding sites have been mapped to CCP7-15 and CCP19-20, whereas the predominant binding to “self” cells via polyanions is via CCP19-20 (41, 42). Interestingly, studies involving the mapping of FH-binding sites on other pathogens such as *Borrelia* spp.*, Neisseria* spp.*, Haemophilus influenzae, Pseudomonas aeruginosa, Candida albicans* and *Staphylococcus aureus* have implicated a common binding footprint for these diverse interaction molecules with either CCP5-7 and/or CCP19-20. There are exceptions to this with the binding site for *Streptococcus pneumoniae, Streptococcus agalactiae* and *Onchocerca volvulus* residing outside these common regions. Using recombinant CCP fragments of FH, we mapped the binding footprint on FH for IAV strains. We found that CCP5-7 and CCP15-20 mediated binding of four different IAV subtypes (**Figure 2**). IAV also bound to the common pathogen interaction sites on FH leaving the regulatory CCP1-4 available for complement inhibitory functions. Further work is required to narrow down the footprint precisely and determine if FH can still inhibit C3 when bound to IAV, and thus, act as a shield from the complement alternative pathway.

Three viral proteins are embedded within the envelope of IAV: haemagglutinin (HA), Neuraminidase (NA) and the ion channel M2. It has been shown previously, using far western blot under reducing conditions, that FH may interact with HA, NA, and the Matrix protein (M1) from IAVs (28). Since HA is the most abundant protein on the surface of influenza viruses, we examined the HA derived from a range of human and avian IAV strains in the context of pseudotyped lentivirus particles, and found that HA alone was able to bind to FH in an ELISA (**Figure 3**). The HA protein of IAV has multiple functions in the viral lifecycle, which include binding to the sialic acid host cell receptor, and enabling attachment and insertion of the fusion peptide into the endosomal membrane to allow viral genome entry into the cell. In addition, it is the primary target of the antibody response of the host, and consequently, can be variable in its sequence. Our mapping of the binding footprint of FH on the HA structure was carried out using overlapping peptides of the HA of both the H1 and H3 subtypes (**Figure 4**). The HA protein of human IAV is processed in two peptides by host trypsin-like proteases, HA1 and HA2, held together by disulphide bonds. We found that FH predominantly bound to peptides located in the HA1 globular head of the HA protein which contains the receptor binding site of IAV. The receptor binding site of HA is formed by two surface loops, the 220-loop (amino acids 225-228) and the 130-loop (amino acids 135-138), and the 190-helix (amino acids 187-194) (43). For both the H1 and H3 HA, we found that FH could bind peptides that correspond to all three sites that make up the receptor binding site, suggesting that FH may occlude the receptor binding site, impeding attachment to target cells and endocytosis of the virus. Interestingly, there was some binding of FH to the membrane proximal stem region of HA for both the H1 and H3 peptide panels although this was more extensive for the H3 HA. The fusion peptide is comprised of the first 25 N-terminal amino acids of the HA2 peptide (amino acids 330-355). The pocket into which the fusion peptide sits and initiates the conformational orientation change of the fusion peptide at low pH is made up of residues at the N-terminal end of HA1 (amino acids in the region of 17) as well as HA2 residues (amino acids spanning the region 380 - 440) (44, 45). FH bound to peptides that encompassed the fusion peptide and the fusion pocket in the H3 HA panel, suggesting likely impairment in the ability of H3 to undergo the conformational change that leads to membrane fusion and release of viral genome into the host cell. In addition, binding of FH to peptides H1-D8 and H3-C8, in close proximity to the HA cleavage site, could interfere with trypsin cleavage of HA and prevent fusion. However, the peptide library does not account for the natural state of the HA protein which forms a trimer, is inserted into the membrane of the virion and can be extensively glycosylated in a strain-specific manner, and therefore, the ability of FH to form these contacts with the natural HA protein as well as the strength of this interaction needs further investigation.

To assess whether the interaction between FH and IAV HA affects life cycle functions of the virus, we carried out infection assays where IAVs were preincubated with FH before being allowed to infect a human pneumocyte cell line, A549 (**Figures 5**, **6**). We examined the ability of the virus to attach to and enter the target cell at early time points and produce viral proteins (nucleoprotein, NP or matrix protein, M1) as well as the ability to complete a whole infectious lifecycle resulting in the release of infectious progenies from the infected cells. For both H1 and H3 IAV subtypes, we found virus entry was impeded at early time points, suggesting that the interaction with the receptor binding site in the HA does abrogate its ability to bind sialic acid. It is also possible that cleavage of HA into HA1 and HA2 by trypsin is affected by FH binding. For the H3N2 subtype, there was a complete blockage of virus entry into cells that was not observed for the H1N1 subtype, possibly due to the additional action of FH on the H3 HA through binding to the fusion peptide, thereby preventing membrane fusion. In the presence of FH, even at the low airway lumen physiological concentration of 5 µg/ml, H3N2 virus was significantly inhibited (**Figure 6**). In contrast, for the H1N1 subtype, after the initial inhibition of virus entry caused by preincubation with FH, the virus replication cycle recovered to show robust infection and release of virus in A549 cells. This suggests that the interaction with the H1 subtype was either of lower affinity and competed off the HA more easily, or the lack of FH binding to the fusion peptide meant that once attachment had successfully occurred, FH posed no impediment to membrane fusion and viral genome release. Although FH bound to the avian IAVs tested, the binding showed no impairment in full virus lifecycle replication in A549 cells at the concentration as high as 100 µg/ml of FH.

We have already described interaction between H1N1 and H3N2 IAV and human FH in a previous study (28). There, we described that concentration of FH had a differential effect on hemagglutination of H1N1 and H3N2 viruses on guinea pig red blood cells (RBC). Indeed, low (2.5 μg/ml), but not high (20 μg/ml), concentration of FH could inhibit hemagglutination of H1N1 viruses on RBC. Conversely, only high concentrations (10 or 20 μg/ml) could inhibit H3N2 hemagglutination. These data suggested that an interaction of FH with HA interferes with sialic acid binding on RBC, but that high concentrations of FH may further promote the interaction of FH/IAV complexes with RBC for H1N1 viruses. Our data suggested that both the virus subtype and the local FH concentration may dictate the final virus infectivity outcome.

Relevance of our findings should be tested *in vivo*. Models of IAV infection in Cfh-deficient mice could be used to address this question (46). Depending on the pathogen studied, different outcomes have been observed in Cfh-deficient mice. For instance, although several different outer surface proteins of *Borrelia burgdorferi*, the causative agent of Lyme disease, have been identified as being able to bind host FH, it has been shown that FH is not essential for mammalian infection by this spirochete (47). In another study, it was demonstrated that Cfh−/− mice with pneumococcal meningitis had increased mortality compared to wild-type mice (48). In humans, FH deficiency is the main cause of atypical hemolytic uremic syndrome as well as kidney and eye diseases. To our knowledge, no correlation between incidence/virulence of influenza A virus infection has been reported in individuals with FH deficiencies or polymorphisms.

In summary, we show that FH can bind diverse IAV strains, including those isolated from avian hosts; this binding is predominantly mediated by interaction with the viral HA protein. We have mapped the interaction footprint and shown that IAV binds to common CCP domains in human FH that other pathogens also bind to evade complement. In addition, the binding to the HA protein by FH occurs in the region of the receptor binding site but additional binding to the fusion peptide is strain-specific. As a result of the strain specific interaction profiles of FH with HA proteins, the impact on viral replication is also strain specific. Further investigation is required to determine if the binding of FH to IAV is an impediment to the virus, or as seems to be the case for H1, H5 and H9 IAV subtypes tested here, there is no restriction offered to the virus by FH binding. In the environment of the respiratory tract, recruitment of locally produced FH can potentially and alternatively aid the survival of IAV by acting as a complement evasion strategy.

## Data availability statement

The original contributions presented in the study are included in the article/**Supplementary Material**. Further inquiries can be directed to the corresponding author.

## Ethics statement

Ethical approval was not required for this study in accordance with the local legislation and institutional requirements because only commercially available established cell lines were used.

## Author contributions

IR: Data curation, Funding acquisition, Investigation, Methodology, Validation, Writing – original draft. EB: Data curation, Formal analysis, Methodology, Software, Writing – review & editing. BN: Conceptualization, Methodology, Supervision, Writing – review & editing. JS: Investigation, Methodology, Resources, Writing – review & editing. AP: Investigation, Project administration, Supervision, Writing – review & editing. MI: Investigation, Methodology, Resources, Writing – review & editing. NT: Investigation, Methodology, Resources, Writing – review & editing. PZ: Data curation, Methodology, Resources, Writing – review & editing. CS: Methodology, Resources, Writing – review & editing. UK: Conceptualization, Resources, Supervision, Writing – original draft. HS: Conceptualization, Funding acquisition, Investigation, Methodology, Project administration, Resources, Supervision, Writing – original draft.

## Supplementary material

Supplementary Figure 1 FH was purified from human plasma and subjected SDS-PAGE under reducing conditions **(A)**, followed by western blot with anti-FH OX24 antibody. Commercial purified FH (Com. FH) used as a control **(B)**. **(C)** Coomassie-stained SDS-PAGE analysis of purified IAV preparations. The six strains of avian and human viruses in the panel were purified by sucrose gradient centrifugation and subjected to a 10% reducing SDS PAGE and stained with Coomassie blue. The locations of the viral proteins are characterised by their expected molecular weights.

Supplementary Figure 2
**(A)** Detection of FH fragments using anti-human FH antisera by western blot. 5 μg of each recombinant fragment - CCP1-4, CCP8-11, CCP11-15, CCP15-18, CCP8-20, and CCP1-7 were subjected to 10% reducing SDS-PAGE, followed by immunoblotting with polyclonal anti-human FH antiserum. Full-length FH was used as a positive control, whilst BSA was used as a negative control (5 μg each). The mobility of size markers is indicated to the left of the gel. Dot blot assay to confirm the solubility of synthetic HA peptides from **(B)** H1N1pdm09 and **(C)** H3N2/99. Each individual peptide (10 amino acids) was blotted on PVDF membrane with concentration of 2.5 μg. The membrane was then probed with IRDye 800CW HRP-conjugated Streptavidin at 1:500 dilution. As a negative control, BAS was used at the same dilution of HA peptide. The Odyssey Imaging System was used to visualize the interaction between the HA peptide and the HRP-conjugated streptavidin.
