# Supplementary material for: Mapping the interaction sites of human and avian influenza A viruses and complement factor H
Source: Front Immunol. 2024 Apr 18;15:1352022. doi: 10.3389/fimmu.2024.1352022 (PMC11064062; doi:10.3389/fimmu.2024.1352022)
Supplement: Supplementary Figure 1 — FH was purified from human plasma and subjected SDS-PAGE under reducing conditions (A), followed by western blot with anti-FH OX24 antibody. Commercial purified FH (Com. FH) used as a control (B). (C) Coomassie-stained SDS-PAGE analysis of purified IAV preparations. The six strains of avian and human viruses in the panel were purified by sucrose gradient centrifugation and subjected to a 10% reducing SDS PAGE and stained with Coomassie blue. The locations of the viral proteins are characterised by their expected molecular weights. [file DataSheet_1.pdf]

Supplementary Material

Mapping the interaction sites of human and avian Influenza A viruses and complement Factor H

Running title: Factor H and influenza A virus interaction.

Iman Rabeeah[1,2], Elizabeth Billington[1], Béatrice Nal[3], Jean-Remy Sadeyen[1], Ansar A. Pathan[2], Munir Iqbal[1], Nigel J. Temperton[4], Peter F. Zipfel[5,6], Christine Skerka[5], Uday Kishore[7,8*], Holly Shelton[1]

[1]Pirbright Institute, Pirbright, Woking, UK.

[2]Biosciences, College of Health, Medicine and Life Sciences, Brunel University London, Uxbridge, UK.

[3]Aix-Marseille Université, CNRS, INSERM, CIML, 13288, Marseille, cedex 9, France

[4]Pseudotype Unit, University of Kent, Chatham, UK.

[5]Leibniz Institute for Natural Product Research and Infection Biology, Jena, Germany.

[6]Friedrich Schiller University, Jena, Germany.

[7]Department of Veterinary Medicine, United Arab Emirates University, Al Ain, U.A.E.

[8]Zayed Centre for Biomedical Sciences, U.A.E. University, Al Ain, U.A.E.

*Corresponding author: uday.kishore@uaeu.ac.ae; ukishore@hotmail.com

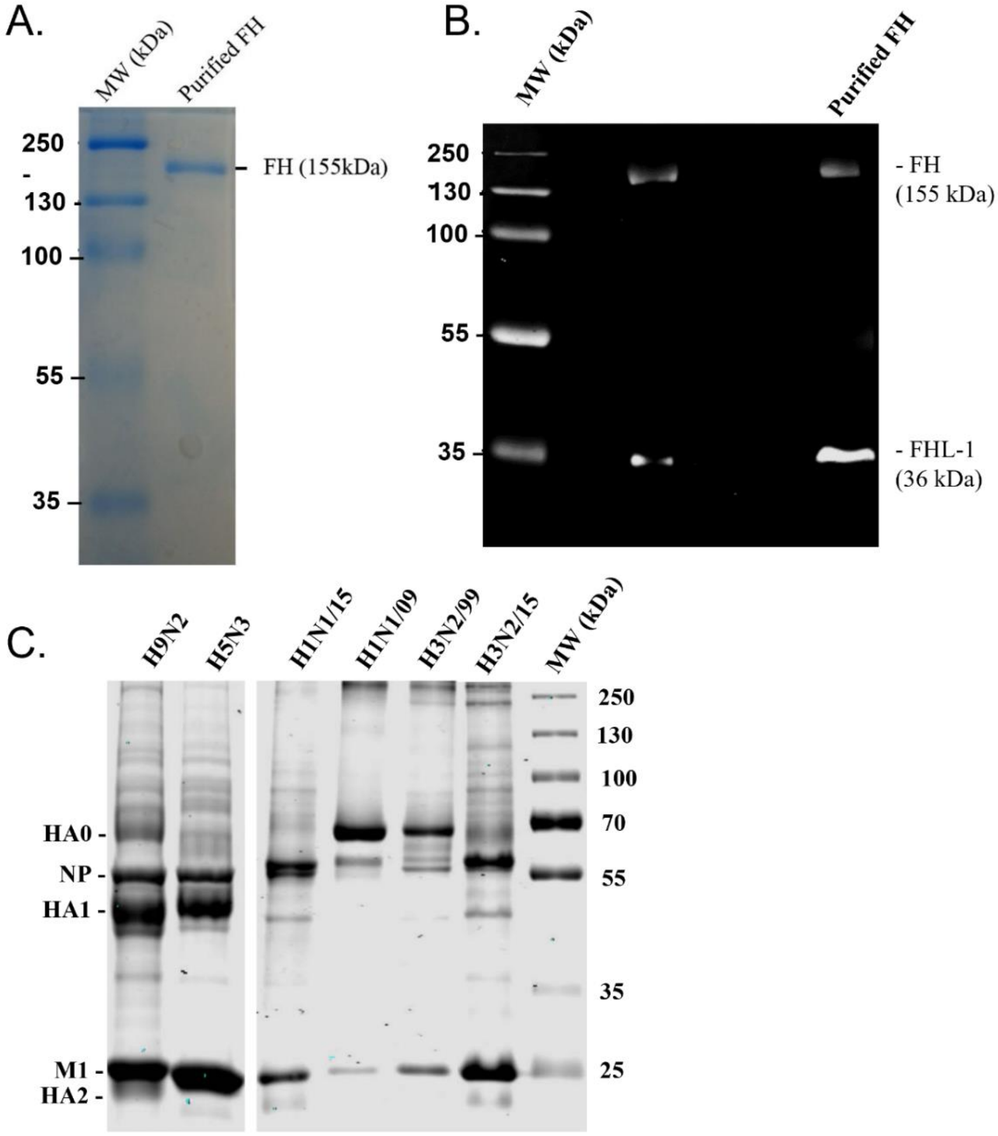

Supplementary Figure 1. FH was purified from human plasma and subjected SDS-PAGE under reducing conditions (A), followed by western blot with anti-FH OX24 antibody. Commercial purified FH (Com. FH) used as a control (B). (C) Coomassie-stained SDS-PAGE analysis of purified IAV preparations. The six strains of avian and human viruses in the panel were purified by sucrose gradient centrifugation and subjected to a 10% reducing SDS PAGE and stained with Coomassie blue. The locations of the viral proteins are characterised by their expected molecular weights.

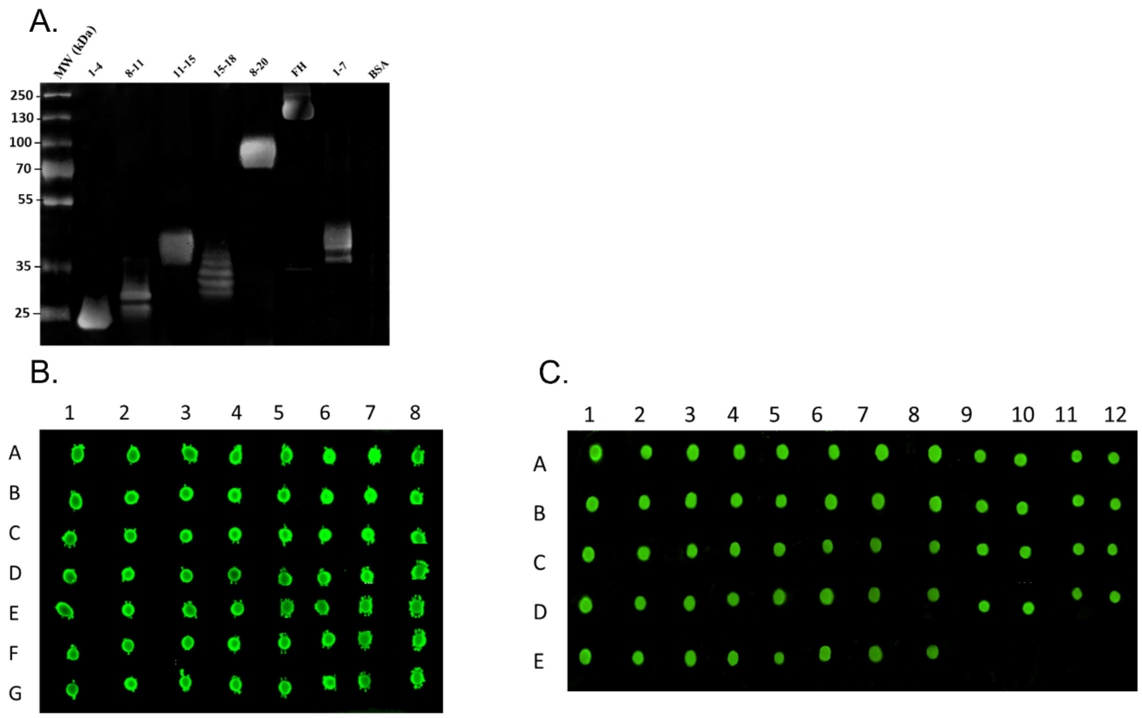

Supplementary Figure 2. (A) Detection of FH fragments using anti-human FH antisera by western blot. 5 µg of each recombinant fragment - CCP1-4, CCP8-11, CCP11-15, CCP15-18, CCP8-20, and CCP1-7 were subjected to 10% reducing SDS-PAGE, followed by immunoblotting with polyclonal anti-human FH antiserum. Full-length FH was used as a positive control, whilst BSA was used as a negative control (5 µg each). The mobility of size markers is indicated to the left of the gel. Dot blot assay to confirm the solubility of synthetic HA peptides from (B) H1N1pdm09 and (C) H3N2/99. Each individual peptide (10 amino acids) was blotted on PVDF membrane with concentration of 2.5 µg. The membrane was then probed with IRDye 800CW HRP-conjugated Streptavidin at 1:500 dilution. As a negative control, BAS was used at the same dilution of HA peptide. The Odyssey Imaging System was used to visualise the interaction between the HA peptide and the HRP-conjugated streptavidin.

Supplementary Table 1: Sequence of H3N2 and H1N1 peptides used in this study.

**H1N1 - A/England/195/09**					
Peptide ID	Sequence*	Amino acid residues	Average OD 450-690 nm	Standard error (±)	Significance value from FH-HA (Adjusted P value)
A1	MKAILVVLLYTFATANADTL	1-20	0.00300	0.00047	0.9991
A2	TFATANADTLCIGYHANNST	10-30	0.13467	0.00072	<0.0001
A3	CIGYHANNSTDTVDTVLEKN	20-40	0.02367	0.00381	0.9814
A4	DTVDTVLEKNVTVTHSVNIL	30-50	0.00567	0.00054	0.9995
A5	VTVTHSVNILEDKHNGKLCK	40-60	0.48500	0.01702	<0.0001
A6	EDKHNGKLCKLRGVAPLHLG	50-70	0.34000	0.00759	<0.0001
A7	LRGVAPLHLGKCNIAGWILG	60-80	1.03867	0.01274	<0.0001
A8	KCNIAGWILGNPECESLSTA	70-90	0.10500	0.00356	<0.0001
B1	NPECESLSTASSWSYIVETS	80-100	0.05933	0.00178	0.0002
B2	SSWSYIVETSSSDNGTCYPG	90-110	0.12667	0.00412	<0.0001
B3	SSDNGTCYPGDFIDYEELRE	100-120	0.06067	0.00196	0.0001
B4	DFIDYEELREQLSSVSSFER	110-130	0.00600	0.00047	0.9996
B5	QLSSVSSFERFEIFPKTSSW	120-140	0.67433	0.00331	<0.0001
B6	FEIFPKTSSWPNHDSNKGVT	130-150	0.05433	0.00072	0.0012
B7	PNHDSNKGVTAACPHAGAKS	140-160	0.50100	0.00984	<0.0001
B8	AACPHAGAKSFYKNLIWLVK	150-170	1.17400	0.02701	<0.0001
C1	FYKNLIWLVKKGNSYPKLSK	160-180	0.08800	0.00330	<0.0001
C2	KGNSYPKLSKSYINDKGKEV	170-190	0.04233	0.00072	0.0539
C3	SYINDKGKEVLVLWGIHHPS	180-200	0.13367	0.00423	<0.0001
C4	LVLWGIHHPSTSADQQSLYQ	190-210	0.02400	0.00047	0.9791
C5	TSADQQSLYQNADAYVFVGS	200-220	0.00733	0.00027	0.9997
C6	NADAYVFVGSSRYSKKFKPE	210-230	0.37233	0.00521	<0.0001
C7	SRYSKKFKPEIAIRPKVRDQ	220-240	0.27167	0.00642	<0.0001
C8	IAIRPKVRDQEGRMNYYWTL	230-250	1.24300	0.00613	<0.0001
D1	EGRMNYYWTLVEPGDKITFE	240-260	0.01067	0.00054	0.9999
D2	VEPGDKITFEATGNLVVPRY	250-270	0.03600	0.00047	0.2339
D3	ATGNLVVPRYAFAMERNAGS	260-280	0.08633	0.00272	<0.0001
D4	AFAMERNAGSGIIISDTPVH	270-290	0.01867	0.00303	0.9987
D5	GIIISDTPVHDCNTTCQTPK	280-300	0.08900	0.00047	<0.0001
D6	DCNTTCQTPKGAINTSLPFQ	290-310	0.04333	0.00072	0.0412
D7	GAINTSLPFQNIHPITIGKC	300-320	0.42900	0.01184	<0.0001
D8	NIHPITIGKCPKYVKSTKLR	310-330	1.06467	0.01658	<0.0001
E1	PKYVKSTKLRLATGLRNVPS	320-340	0.00400	0.00094	0.9993
E2	LATGLRNVPSIQSRGLFGAI	330-350	0.00300	0.00047	0.9991
E3	IQSRGLFGAIAGFIEGGWTG	340-360	0.00567	0.00027	0.9995
E4	AGFIEGGWTGMVDGWYGYHH	350-370	0.02700	0.00249	0.8539
E5	MVDGWYGYHHQNEQGSGYAA	360-380	0.00700	0.00082	0.9997
E6	QNEQGSGYAADLKSTQNAID	370-390	0.00500	0.00000	0.9994
E7	DLKSTQNAIDEITNKVNSVI	380-400	0.00633	0.00119	0.9996

E8	EITNKVNSVIEKMNTQFTAV	390-410	0.02233	0.00144	0.9858
F1	EKMNTQFTAVGKEFNHLEKR	400-420	0.00700	0.00047	0.9997
F2	GKEFNHLEKRIENLNKKVDD	410-430	0.00367	0.00054	0.9993
F3	IENLNKKVDDGFLDIWTYNA	420-440	0.00367	0.00027	0.9993
F4	GFLDIWTYNAELLVLLENER	430-450	0.00500	0.00082	0.9994
F5	ELLVLLENERTLDYHDSNVK	440-460	0.00567	0.00191	0.9995
F6	TLDYHDSNVKNLYEKVRSQL	450-470	0.01233	0.00072	0.9997
F7	NLYEKVRSQLKNNAKEIGNG	460-480	0.00733	0.00054	0.9997
F8	KNNAKEIGNGCFEFYHKCDN	470-490	0.03733	0.00242	0.1778
G1	CFEFYHKCDNTCMESVKNGT	480-500	0.00733	0.00027	0.9997
G2	TCMESVKNGTYDYPKYSEEA	490-510	0.00800	0.00047	0.9998
G3	YDYPKYSEEAKLNREEIDGV	500-520	0.00767	0.00027	0.9997
G4	KLNREEIDGVKLESTRIYQI	510-530	0.00767	0.00098	0.9997
G5	KLESTRIYQILAIYSTVASS	520-540	0.00600	0.00047	0.9996
G6	LAIYSTVASSLVLVVSLGAI	530-550	0.00533	0.00027	0.9995
G7	LVLVVSLGAISFWMCSNGSL	540-560	0.00467	0.00072	0.9994
G8	SFWMCSNGSLQCRICI	550-566	0.18567	0.00191	<0.0001
A1 - FH	MKAILVVLLYTFATANADTL	1-20	0.00433	0.00072	0.9993
FH - HA	No peptide		0.00967	0.00423	
**H3N2 - A/Hong Kong/ 1774/99**					
Peptide ID	Sequence*	Amino acid residues	Average OD 450-690 nm	Standard error (±)	Significance value from FH-HA (Adjusted P value)
A1	MKTIIALSYIFCMVLGQDLP	1-20	0.09867	0.00425	0.2054
A2	FCMVLGQDLPGKGNNTATLC	10-30	0.35800	0.00535	<0.0001
A3	GKGNNTATLCLGHHAVPNGT	20-40	1.13300	0.01650	<0.0001
A4	LGHHAVPNGTLVKTITDDQV	30-50	0.07100	0.00094	0.9989
A5	LVKTITDDQVEVTNATELVQ	40-60	0.06633	0.00233	0.9994
A6	EVTNATELVQNLSMGKICSN	50-70	0.42833	0.00453	<0.0001
A7	NLSMGKICSNPHRILDGANC	60-80	0.87167	0.01240	<0.0001
A8	PHRILDGANCTLIDALLGDP	70-90	0.66833	0.00475	<0.0001
A9	TLIDALLGDPHCDGFQNEKW	80-100	0.56467	0.00608	<0.0001
A10	HCDGFQNEKWDLFIERSKAF	90-110	0.48100	0.00572	<0.0001
A11	DLFIERSKAFSNCYPYDVPE	100-120	0.63300	0.00822	<0.0001
A12	SNCYPYDVPEHASLRSLIAS	110-130	0.33133	0.00599	<0.0001
B1	HASLRSLIASSGTLEFVNES	120-140	0.24167	0.01570	<0.0001
B2	SGTLEFVNESFNWTGVTQNG	130-150	0.06433	0.00072	0.9996
B3	FNWTGVTQNGGSNACKRGPD	140-160	0.84033	0.00722	<0.0001
B4	GSNACKRGPDSSFFSRLNWL	150-170	1.30367	0.00662	<0.0001
B5	SSFFSRLNWLYKSGNTYPML	160-180	0.08100	0.00082	0.9657
B6	YKSGNTYPMLNVTMPNSDGF	170-190	0.12567	0.00072	0.0009
B7	NVTMPNSDGFDKLYIWGVHH	180-200	0.11933	0.00196	0.0042
B8	DKLYIWGVHHPSTDREQINL	190-210	0.11967	0.00191	0.0039
B9	PSTDREQINLYVQASGKITV	200-220	0.09900	0.00094	0.1959

B10	YVQASGKITVSTKRSQQTII	210-230	0.10533	0.00152	0.0719
B11	STKRSQQTIIPNVGSRPWVR	220-240	1.02133	0.00576	<0.0001
B12	PNVGSRPWVRGLSSRISIYW	230-250	0.85233	0.01664	<0.0001
C1	GLSSRISIYWTIVKPGDILI	240-260	0.10667	0.00722	0.0569
C2	TIVKPGDILIISSNGNLIAP	250-270	0.12100	0.00455	0.0028
C3	ISSNGNLIAPRGYFKVHTGK	260-280	0.45900	0.00957	<0.0001
C4	RGYFKVHTGKSSIMRSDAPI	270-290	0.11667	0.00633	0.0076
C5	SSIMRSDAPIETCSSECITP	280-300	0.71300	0.00579	<0.0001
C6	ETCSSECITPNGSIPNDKPF	290-310	0.70133	0.03295	<0.0001
C7	NGSIPNDKPFQNVNKITYGA	300-320	0.53233	0.00472	<0.0001
C8	QNVNKITYGACPKYVKQNTL	310-330	1.29367	0.02273	<0.0001
C9	CPKYVKQNTLKLATGMRNIP	320-340	0.31800	0.00776	<0.0001
C10	KLATGMRNIPEKQTRGIFGA	330-350	0.26433	0.01355	<0.0001
C11	EKQTRGIFGAIAGFIENGWE	340-360	0.11700	0.00680	0.0071
C12	IAGFIENGWEGMVDGWYGFR	350-370	0.09633	0.00768	0.2815
D1	GMVDGWYGFRHQNSEGTGQA	360-380	0.10733	0.00566	0.0504
D2	HQNSEGTGQAADLKSTQAAI	370-390	0.08733	0.00472	0.7192
D3	ADLKSTQAAINQINGKLNRV	380-400	0.12333	0.00378	0.0016
D4	NQINGKLNRVIEKTNEKFHQ	390-410	0.10700	0.00330	0.0535
D5	IEKTNEKFHQIEKEFSEVEG	400-420	0.09033	0.00384	0.5561
D6	IEKEFSEVEGRIQDLEKYVE	410-430	0.10933	0.00562	0.0346
D7	RIQDLEKYVEDTKIDLWSYN	420-440	0.10667	0.00347	0.0569
D8	DTKIDLWSYNAELLVALENQ	430-450	0.14400	0.01159	<0.0001
D9	AELLVALENQHTIDLTDSEM	440-460	0.09567	0.00401	0.3067
D10	HTIDLTDSEMNKLFEKTRKQ	450-470	0.12567	0.00828	0.0009
D11	NKLFEKTRKQLRENAEDMGN	460-480	0.09900	0.00340	0.1959
D12	LRENAEDMGNGCLKIYHKCD	470-490	0.95467	0.01606	<0.0001
E1	GCLKIYHKCDNSCIDSIRNG	480-500	0.72567	0.00504	<0.0001
E2	NSCIDSIRNGTYDHNEYRDE	490-510	1.01500	0.00283	<0.0001
E3	TYDHNEYRDEALNNRFQIKS	500-520	0.12700	0.00245	0.0006
E4	ALNNRFQIKSVELKTGYKDW	510-530	0.14267	0.00072	<0.0001
E5	VELKTGYKDWILWISFAISC	520-540	0.17267	0.00393	<0.0001
E6	ILWISFAISCFLLCVVWLGF	530-550	0.06633	0.00072	0.9994
E7	FLLCVVWLGFIMWACQKGNI	540-560	0.08067	0.00072	0.9698
E8	IMWACQKGNIRCNIC	550-565	0.65233	0.00519	<0.0001
A1 - FH	MKTIIALSYIFCMVLGQDLP	1-20	0.05933	0.00027	>0.9999
FH - HA	No peptide		0.06033	0.00119	

*Each Peptide had a N-terminal biotin tag and at the C-terminus an amide.
